# The Polymorphism of Drugs: New Approaches to the Synthesis of Nanostructured Polymorphs

**DOI:** 10.3390/pharmaceutics12010034

**Published:** 2020-01-01

**Authors:** Dmitry Chistyakov, Gleb Sergeev

**Affiliations:** 1 Belozersky Institute of Physico-Chemical Biology, Lomonosov Moscow State University, 119992 Moscow, Russia; 2 Department of Chemistry, M. V. Lomonosov Moscow State University, 119991 Moscow, Russia; gbs28@rambler.ru

**Keywords:** polymorphism, poorly soluble drug, cryotemperature, cryosynthesis, nanoparticles

## Abstract

Among the significant problems of modern pharmacology are the low solubility and bioavailability of drugs. One way to resolve this problem is to obtain new polymorphic forms of drugs with improved physicochemical properties. Various approaches have been developed with this aim, including the preparation of co-crystals, the use of nanoparticles, or the use of compounds in the form of a salt. A promising direction in pharmacology concerns the production of new stable polymorphic structures. In this mini-review, we consider certain aspects of drug polymorphism, methods for the synthesis of polymorphs, and the stability, size, and transformation of crystalline polymorphs. Moreover, we summarize our results from several studies demonstrating the problems associated with the synthesis of new polymorphous modifications based on inert gases and cryotemperatures. The results indicate that the problems specific to drug polymorphisms have only been partly resolved, are of current interest, and require further development.

## 1. Introduction

The majority of present-day medicinal substances are poorly soluble in water, which reduces their bioavailability and makes their practical application difficult [1,2]. Many drugs are solid organic substances, which additionally complicates their application. The methods used for improving the solubility, dissolution rate, and bioavailability of solid medicinal substances are being developed and improved. In the last two decades, in order to enhance the solubility and bioavailability of drugs, in addition to drugs in the form of salts [2], attention has been focused on the preparation of solid amorphous dispersions [3,4], dispersions containing nanocrystals [5,6], and the synthesis of co-crystals [7,8]. Additionally, when resolving the problem of improving the bioavailability, the polymorphism phenomenon, which is defined as the possibility for the original molecule to exist in two or more crystal states, plays an important role [9,10,11].

The higher solubility and bioavailability of polymorphous modifications are associated with the formation of crystals that change the properties of original compounds [11]. Polymorphous structures differ with regard to their physicochemical and pharmacological properties. They also have differences in terms of melting points, solubility, dissolution rate, lattice structure, hardness, stability, and certain other parameters [11].

It was shown that polymorphism is characteristic to 25% of hormones, 60% of barbiturates, and 70% of sulfamides [12]. The synthesis of a new polymorphic modification may be a reason to obtain a patent for the drug used [13,14]. The US Food and Drug Administration (FDA) notes the importance of detecting polymorphic forms of drugs, and also emphasizes the importance of comprehensive control over the polymorphs at different stages of product development [15].

As soon as general problems of drug polymorphism research have been reported in detail in recent reviews [9,16,17], etc., we briefly summarize the main problems that occur in such investigations. The report is focused on the approaches of obtaining new polymorphic forms as nanocrystals.

## 2. Certain Aspects of Drug Polymorphism

Currently to obtain polymorphic forms of drugs various methods are used including single and multi-solvent crystallization, lyophilization, sublimation, spray drying, sublimation, grinding, etc. [11,18]. 

Polymorph properties should include the morphology of crystals, their crystallographic parameters, the size of particles, and the solubility and dissolution rate of each structure. The listed parameters, as well as the physicochemical properties of the original substance should be controlled for dispersions and suspensions involving polymorphs.

If thermodynamics assume the dominant role in the synthesis of polymorphs, stable structures with the lowest free energy are formed. If the kinetics dominate, metastable polymorphous structures with larger free energy are formed [19,20]. Therefore, control over thermodynamic and kinetic parameters during the synthesis of a polymorphous compound is necessary in both fundamental and applied studies. The use of polymorphous structures is complicated by the fact that, for many of them, we do not know their form and unit cell parameters. Another important factor concerning different polymorphs is the difference in their physical properties such as powder property, melting point, enthalpy of fusion, dissolution behavior, etc. [10]. 

The morphology of polymorphs plays a key part in their application; hence, much importance is attached to elucidating the structure of polymorphous modifications. Almost all microscopy options, X-ray single crystal diffraction, solid-state nuclear magnetic resonance, IR spectroscopy including terahertz (3–100 cm^−1^), Raman spectroscopy, and differential scanning calorimetry, are used to study and identify polymorphs of drugs [13,21].

Drug polymorphism requires control over all stages of synthesis, application, and storage. An incident involving the anti-HIV/AIDS drug ritonavir highlighted the need to increase control over drug polymorphism and prompted companies and scientists to commence the comprehensive screening of polymorphous modifications [22,23,24]. Time has shown that the control over the synthesis of new drug polymorphs and the possible transformations of the already-used drug version are still significant problems [17,25].

## 3. Polymorphism and Nanocrystals

An increase in the solubility and dissolution rate of crystalline preparations can be achieved by their micro- and nanonization. In this case, the resulting nanocrystals, as well as crystals of ordinary sizes, have different polymorphic forms with different properties.

### 3.1. Synthesis of Nanocrystals

The method used for the synthesis of medicinal polymorphous substances in the form of nanocrystals is determined by the properties of the original substance. The technologies are divided into four main groups: “top-down” and “bottom-up” (or descending and ascending processes), combined versions, and the chemical synthesis [26]. The top-down technologies require a large amount of energy and a long time for synthesizing nanoparticles. The use of top-down methods is usually limited to the preparation of particles measuring between 150 and 300 nm. The bottom-up technologies are often assumed to include various processes of precipitation from oversaturated solutions [27]. Keen attention has been paid to particles measuring less than 100 nm [28]. For such particles, unusual physical properties have been revealed, in particular, the ability to penetrate various biological barriers [27]. Until recently, various polymorphous modifications of drugs were most often prepared by evaporating of solvent or by melt cooling [13]. The synthesis of Form II of a steroid neurohormone progesterone and the identification of its crystal structure involved using more than 10 different solvents [29,30]. The decoding of the crystal structure of progesterone (II) was repeatedly cited for 50 years [31]. The most reliable result was obtained by co-crystallization of progesterone with the addition of 1% of pregnenolone with a much similar structure [32]. 

When using polymorphous drugs, it is always necessary to take into account the presence of different forms with different properties and select the form suitable for the problem to be resolved. There are versions where a metastable structure has advantages over a stable structure. To convert solid polymorphous structures into nanoparticles, a considerable amount of time is often required. To produce particles with the size of 100 nm and smaller by homogenization under high pressure, several cycles should be performed [33]. The morphology of synthesized polymorphous particles also depends on the methods used. The synthesis of polymorphous nanoparticles can be carried out with the use of spray or sublimation drying technologies, as well as in combination with precipitation. Combining the methods for the synthesis of nanoparticles with their subsequent molecular assembly allows for polymorphous nanocrystals of different forms to be obtained. The changes in the morphology of particles affect their subsequent transformations. For instance, preliminary spray drying of samples leads to destruction during subsequent homogenization under high pressure [33]. When synthesizing drugs, thermodynamically stable forms are used, which avoids possible transformations during their scaling, synthesis, and storage [34]. It was shown that the chemical and physical stability of these dispersions remains unchanged [35]. It was noted that the process of homogenization under high pressure can induce the transformation of polymorphous structures [36]. It is also noteworthy that the competition between the structure and the size of nanocrystals may affect the solubility of polymorphous modifications. A case where the structure of nanocrystals affected their solubility more strongly than their size was considered in [37]. 

### 3.2. Stability, Size, and Transformation of Nano-Crystalline Polymorphs

At present, there is a focus on the influence of the physicochemical properties of original compounds on the synthesis and stability of polymorphous nanocrystals, suspensions, and dispersions involving their participation. The effect of various factors on certain technologies for the synthesis of nanocrystals was analyzed in a review [26]. Aspects of the commercial use of nanocrystals and nanosuspensions in pharmacology and medicine were discussed in [38]. The requirements for assessing the polymorphous modifications were formulated. It was found that changes in their form must not affect the quality and bioavailability of the product; it must be ensured that no transition from one polymorphous structure to another occurs during storage; appropriate methods for of analysis and sampling as well as exercising control over samples must be selected in order to rule out transforming the polymorphous modifications of drugs before their use by patients.

Earlier, it was shown that, by changing the temperature of the experiment, the substance concentration, and the solvent, it is possible to obtain several polymorphous modifications to different structures in a single solvent [39]. The synthesis of precipitated particles depends on the solvent–antisolvent combination, the concentrations of components, and the conditions of their mixing. It was shown that the precipitation and stabilization of fine crystals play an important role in their further transformation into coarser nanocrystals [27]. The polymorph morphology can affect not only the particle size but also the stabilization of crystals. For example, the temperature of the experiment determines the viscosity of the medium and the transport of dissolved molecules. The latter phenomenon can change the morphology of final nanocrystals [40]. It was found that the nature of added stabilizers also affects the morphology of nanoparticles. The synthesis of a certain polymorphous structure depends on the solvent–antisolvent–stabilizer combination and the degree of oversaturation. During precipitation, different solvents can change the polymorphous structure, the size, and the stability of particles. Thus, the appropriate choice of precipitation parameters is a necessary condition for the synthesis of polymorphous modifications with definite morphology. Kinetically-stable polymorphous modifications have different storage times; hence, their use requires the development of methods that would help to extend their lifetime. To this extent, the synthesis of new, stable nanostructures is of interest in connection with polymorphous drug modifications.

In the review by Cruz-Cabeza et al., based on experimental and theoretical data as well as crystallographic data from the Cambridge database, the general problems associated with polymorphism in various classes were considered [25]. The analysis of the lattices of various polymorphous structures was carried out using the density functional method. Attention was centered on salts and co-crystals, with a special section devoted to drug polymorphism. Problems with the general and theoretical screening of polymorphous compounds were also mentioned. The comprehensive study of the problem of screening and the analysis of the data obtained by different companies allowed the authors to conclude that the problem concerning the universal screening of polymorphous drugs has only been partly resolved and thus remains an important and topical issue. Based on calculation methods and computer-assisted analyses combined with experimental data, it is difficult to predict whether a given substance can produce solid polymorphous modifications to crystallization. Each drug is individual, and it is equally difficult to predict whether the substance can simply crystallize.

## 4. Approaches for the Synthesis of New Polymorphic Nanostructures

In synthesizing and studying drug polymorphism, new possibilities have been opened up in light of an original strategy proposed in [41,42,43,44,45]. The developed technology is based on the conversion of the initial substance into a gaseous state in the course of its sublimation within the flow of an inert gas-carrier, followed by condensation on contact with a surface cooled by liquid nitrogen.

### 4.1. Effect of Inert Gases on the Synthesis of New Polymorphous Nanostructures

The technique that involves the use of inert gases and employs cryotemperatures was initially aimed at the synthesis of nanoparticles; however, the very first experiments produced new unexpected results. For the tranquilizer phenazepam (7-bromo-1,3-dihydro-5-(2-chlorophenyl)-2*H*-1,4-benzodiazepin-2-one), sublimation in the nitrogen flow was followed by condensation, in turn producing a new crystal form. Its analysis was performed by applying the powder X-ray diffraction technique. A comparison with the known literature data revealed that this is a new polymorphous form of phenazepam, while the lattice parameters were determined [41]. In this new polymorphous structure, two benzene rings form a dihedral angle of 86.2(3)°, whereas, in pharmaceutical phenazepam, this angle is 75.4(2)°. In both polymorphs, the molecules are bonded by intermolecular hydrogen bonds N–H···O to form centrosymmetrical dimers. In the crystal structure of the new phenazepam form, the non-classical intermolecular hydrogen bond C–H···O connects these dimers into layers. Transmission electron microscopy has shown that the crystals of this new form have an average size of 50 nm. Thus, the developed strategy has produced not only a new polymorphous structure but also new forms of nanocrystals.

**Figure 1:**
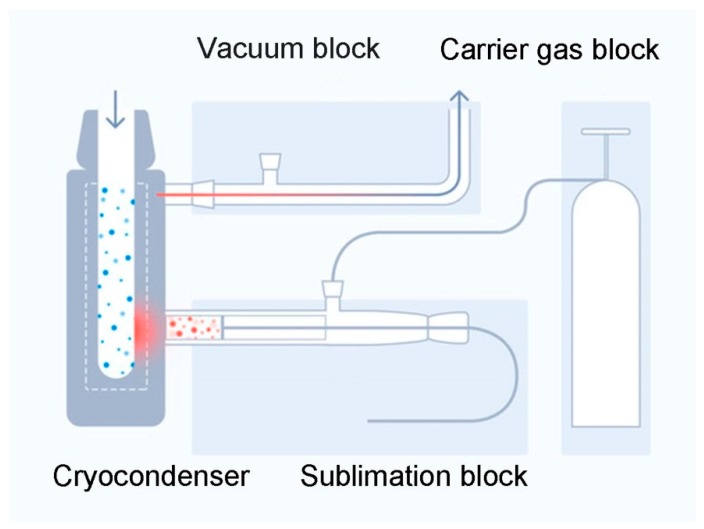
Nanostructure synthesis block-scheme (adapted from [44]).

The further development of the cryochemical strategy, as exemplified by the steroid neurohormone dehydroepiandrosterone (DHEA), led to the disclosure of new effects [45]. The transition of this neurohormone to the gas phase, together with inert gas and the interaction between the mixed flow and a surface cooled by liquid nitrogen, produced nanoparticles and led to their transformation into new polymorphous nanocrystals [43,45]. The setup for the synthesis of nanocrystals (Figure 1) was described in [45]; two new polymorphous nanostructures that can only be formed in the presence of inert gas were obtained [45]. The crystallographic parameters of these new DHEA structures can be found in [43].

Thus, the involvement of gases carriers in this process led to the discovery of the phenomenon of the inert gas effect as a new factor in cryochemical synthesis and in the transformation of nanoparticles synthesized from the initial compound into new polymorphous nanocrystals. For DHEA, six polymorphous anhydrous modifications are known [46]. Only three forms (Forms I (FI), II (FII) and VI (FVI)) were crystallographically defined. The use of inert gases and cryotemperatures produced two new forms (Forms VII (FVII) and VIII (FVIII)). The crystallographic structure of FVII was decoded by means of the powder X-ray diffraction method. Form II, obtained earlier, was also identified. In the previously known polymorphous modifications, hydrogen bonds are formed by hydroxyl and carbonyl oxygen, enabling the head–tail interaction. The new polymorphous nanostructure FVII takes the form of a spiral chain, in which DHEA molecules form hydrogen bonds exclusively through the oxygen atoms of hydroxyl groups. Form III has a spiral structure with two chains connected by hydrogen bonds. In one of these chains, the bonds are formed by hydroxyl groups, while, in the other chain, they are formed by carbonyl groups [43]. Using the density functional method, the lattice energy was assessed for the structure of the Forms I, II, II (FIII), and VII. It was shown that the lattice energy of different forms differs insignificantly within the range of 1 kcal [43]. A slight difference in the lattice energy of DHEA polymorphous modifications can lead to mixtures of different forms and complicate their separation by varying the temperature. The morphology and formation of nanocrystals obtained under comparable conditions are determined by the nature of the incondensable inert gas [45]. The highest yield and selectivity of new nanocrystals FVII and FVIII were obtained with the use of carbon dioxide. The selectivity was 70% for FVII and 30% for FVIII. The results for helium and nitrogen showed that the yields of the new forms, FVII and FVIII, decreased and other forms, FII and FIII, appeared [45]. The size of nanocrystals obtained depended weakly on the nature of the gas carrier, whereas the nature of the original medicinal substance had a stronger effect on the size. Under comparative conditions with a condenser temperature of −196 °C and the use of nitrogen as the gas carrier, the average particle size was 50 nm for phenazepam and 120 nm for DHEA. Thus, the results given above demonstrate that the morphology of polymorphs can be regulated by using the dynamic mode, cryosynthesis, and various inert gases.

It can be assumed that the proposed approach can be scaled to obtain a larger volume of substances. The laboratory setup, the scheme of which is shown in Figure 1, is capable of producing up to 1 g of a modified substance per day. 

The cryochemical technology was used in the synthesis of nanoparticles of piroxicam [47], androstenediol [48], phenazepam [41], and DHEA [45]. Experiments with various molecules revealed the limitations for substances suitable for transformation. These are substances that have an oxygen, nitrogen, and sulfide group. Substances that do not decompose on heating. They have a melting point of 100–250 °C and can form hydrogen bonds. These substances should not have groups that can interact in the gas phase. The molecular weight of the substance should not be too large, we had a positive experience with the application of the cryosynthesis method in the case of substances with a molecular weight of 400 g/mol. The proposed strategy allowed to synthesize new polymorphic nanostructures for phenazepam and DHEA. Currently, further fundamental physical and chemical studies are needed to predict which molecules with new polymorphic structures can be obtained.

### 4.2. Effect of Temperature on the Size, Stability, and Transformations of New Nanostructures

Small amounts of CO_2_ can be captured by the condenser at a temperature of −196 °C. It was assumed that the linear CO_2_ molecule can influence the structural formation of FVII [45]. In experiments with a condenser temperature of −78 °C, samples were prepared in the same way as at a temperature of −196 °C. The experiments at these two temperatures (−196 and −78 °C) have shown that the phase composition is independent of the temperature. In our opinion, this suggests the absence of any effect of CO_2_ in the formation of the nanocrystal structure in the nucleation, growth, and formation stages of the final product. The invariance of the phase composition at temperatures of −196 and −78 °C additionally confirmed the important role played by the preparation of samples in the gas phase in the crystallization and cryochemical synthesis of nanocrystals. Experiments at −78 °C also involved possible intermolecular interactions between CO_2_ and DHEA, which should increase as the temperature decreases. An increase in cryotemperature by more than 100 °C changed the crystal size from 90 nm at −196 °C to 335 nm. Hence, the size of new polymorphous modifications can be controlled by changing the temperature of the experiment. 

Studying the stability of DHEA Forms VII and VIII showed that, at room temperature, the mixture was stable for up to two months and, at −5 °C, it was stable for 12 months. The high level of stability in new forms is determined by the synthetic conditions and the presence of activation barriers between individual phase structures. The joint consideration of data obtained at cryotemperatures with those obtained at high temperatures showed that it is possible to carry out controlled syntheses in order to produce samples either with the same structure and different sizes or with the same size but different structures. When stored under standard conditions, the new nanoform of phenazepam was stable for more than two years [42]. By determining the solubility and dissolution rate, compared with the commercial form, the solubility of the new nanoform in water is higher by more than 30% and its dissolution rate increases by a factor of 4.9 [42]. 

The results obtained offer new possibilities for the controlled synthesis of, and investigation into, the effect of morphology and size of polymorph nanocrystals. Experiments on the effect of temperature on the stability of phenazepam, mixture of DHEA FVII and FVIII, in combination with the results from Section 4.1, extended and refined the strategy developed. Conditions are formulated for exercising control over the synthesis, structure, stability, transformations, and size of the polymorphous modifications of drugs by using cryochemistry and inert gases carriers. The original molecules are transferred into the gas phase by sublimation in the flow of a heated inert gas carrier. 

Interaction between two gases involves inelastic collisions of gas molecules and their flows with sublimator walls. These processes are accompanied by an exchange of energy between molecules. The change in energy is determined by the temperature of the experiment and the nature, mass, rate, and heat capacity of the initial substance. As a result, a mixed stationary gas flow is organized in which the molecules of the initial substance obtain a certain energy. At the stage when the mixed flow of molecules collides with the cryocondenser surface, the temperature of the hot flow sharply drops. This is accompanied by the heterogeneous formation of a large number of fine nuclei. In the next stage, we observe the dynamic growth of nanoparticles, molecular assembling, and the formation of stable nanocrystals with the new polymorphous structure.

The experiments carried out at different temperatures extended the potential for the synthesis of polymorphous compounds. It was found that cryotemperature affects the stability of nanocrystals although it has no substantial effect on the morphology of the polymorphous structure. Combining experiments with the use of different inert uncondensed gases, with a wide temperature interval from low (−196 °C) to high (120 °C), produced new polymorphous nanostructures for the tranquilizer phenazepam and the steroid neurohormone DHEA. The totality of the results shows that, in the synthesis of new polymorphous compounds, inert gases and their nature are the main contributors in the phase composition and the selectivity of synthesized polymorphs. The above result can be interpreted, based on the principle known in chemistry as the relationship between selectivity and activity. According to this principle, the higher the activity, the lower the selectivity and vice versa. From our point of view, when CO_2_ is used as the inert gas, the activity of DHEA molecules is lower, which results in the higher selectivity of Form VII [45]. Crystallization at cryotemperatures determines the size of nanocrystals and is responsible for their stability. The use of high temperatures leads to the transformation of the cryopolymorphous structure of DHEA into stable nanosamples of different sizes and compositions. Thus, the proposed strategy allows for new polymorphous nanocrystals of different sizes and structures to be synthesized from the original molecule.

## 5. Conclusions

Both individual scientists and pharmaceutical companies have emphasized the increasing interest in drug polymorphism and the synthesis of polymorphous crystals with the aim of increasing their solubility and bioavailability. The appearance of new methods and approaches to the synthesis of polymorphous nanostructures, such as cryonanosynthesis, the development of methods for the theoretical and computational prediction of new structures, and comprehensive studies on drug polymorphs will lead to the production of new, safe, and efficient drugs.
